# Resveratrol Improves Liver Steatosis and Insulin Resistance in Non-alcoholic Fatty Liver Disease in Association With the Gut Microbiota

**DOI:** 10.3389/fmicb.2021.611323

**Published:** 2021-02-23

**Authors:** Fan Du, Rongfeng Huang, Dan Lin, Yuying Wang, Xiaohuang Yang, Xiaoyun Huang, Biyun Zheng, Zhixin Chen, Yuehong Huang, Xiaozhong Wang, Fenglin Chen

**Affiliations:** ^1^Department of Gastroenterology, Fujian Medical University Union Hospital, Fuzhou, China; ^2^Fujian Medical University Cancer Center, Fujian Medical University, Fuzhou, China

**Keywords:** resveratrol, non-alcoholic fatty liver disease, gut microbiota, anaerobic culture, insulin resistance

## Abstract

Resveratrol (RSV) is a potential alternative therapy for non-alcoholic fatty liver disease (NAFLD) that has been evaluated in many clinical trials, but the mechanisms of RSV action have not been fully elucidated. Recent studies suggested that the gut microbiota is an important RSV target; therefore, we speculated that the gut microbiota might mediate the beneficial effects of RSV in NAFLD. To verify this hypothesis, we established a high-fat diet (HFD)-induced NAFLD mouse model, which was subjected to RSV gavage to evaluate the therapeutic effects. We observed that RSV reduced liver steatosis and insulin resistance in NAFLD. RSV significantly changed the diversity and composition of the gut microbiota according to 16S rRNA sequencing. Gut microbiota gene function prediction showed that the enrichment of pathways related to lipid and glucose metabolism decreased after RSV treatment. Furthermore, correlation analysis indicated that the improvements in NAFLD metabolic indicators were closely related to the altered gut microbiota. We further fermented RSV with the gut microbiota *in vitro* to verify that RSV directly affected the gut microbiota. Our data suggested that the gut microbiota might be an important target through which RSV exerts its anti-NAFLD effect.

## Introduction

Non-alcoholic fatty liver disease (NAFLD) is a clinical pathological syndrome characterized by excessive fat deposition in liver cells in the absence of excessive alcohol consumption and other specific liver damaging factors (Wong et al., 2018). This disease can be reversed at an early stage, but without intervention, it may develop into cirrhosis or liver cancer in the later stage and seriously endanger life and health (Romero-Gomez et al., 2017). Although there are currently several drug trials targeting NAFLD, no approved medicine has yet been developed. The current treatments are based on calorie restriction and exercise, but patient compliance is unsatisfactory and difficult to maintain. Therefore, it is necessary to find drugs with definite therapeutic effects on NAFLD (Review et al., 2014; Sumida and Yoneda, 2018).

Resveratrol (RSV) is a non-flavonoid polyphenol compound that is naturally synthesized by plants (Huminiecki and Horbanczuk, 2018). Previous studies have suggested that RSV can improve various metabolic diseases via multiple mechanisms, such as effects on oxidative stress, inflammation, and adipokines (Chen et al., 2017; Chaplin et al., 2018). RSV is regarded as a candidate drug for NAFLD. However, not all clinical studies have reported beneficial results (Faghihzadeh et al., 2015; Elgebaly et al., 2017), and limited evidence has been found to explain the disparities. Consequently, a comprehensive evaluation of the mechanism of RSV is critical to explain the disparities between studies and facilitate its use as a candidate therapeutic drug for NAFLD.

Considering the low bioavailability of RSV in the intestine, a large part of unmetabolized RSV arrives in the colon and interacts with the gut microbiota, therefore, the gut microbiota might be a potential target (Walle, 2011). The gut microbiota consists of the wide variety of parasitic microorganisms in the human intestinal tract and shows dynamic changes (Sekirov et al., 2010). RSV can be modified by the gut microbiota via hydrogenation and produce dihydroresveratrol that promote its absorption in the intestine (Juan et al., 2010; Walle, 2011). In turn, the interaction of RSV and the gut microbiota can change the composition of the gut microbiota (Chaplin et al., 2018). In addition, previous studies have shown that changes in the gut microbiota are an important factor in the progression of NAFLD (Saltzman et al., 2018; Aron-Wisnewsky et al., 2020). The gut microbiota can interact with the liver through the gut-liver axis, and the tight junctions within the gut form an important barrier in the gut-liver axis that prevents harmful metabolic products from entering the liver and maintaining its function (Milosevic et al., 2019), indicating that tight junctions may be a potential target of the gut microbiota. Damage to tight junctions will causes increases in inflammation and oxidative stress (Zhang et al., 2000; Kim et al., 2012), and excessive inflammation can jeopardize insulin signaling pathways and fatty acid metabolism, subsequently aggravating insulin resistance and liver steatosis and ultimately exacerbating NAFLD (Tilg and Moschen, 2008; Leavens and Birnbaum, 2011). Furthermore, insulin resistance can promote the progression of other metabolic diseases, such as type 2 diabetes and cardiovascular disease, which endanger health (Asrih and Jornayvaz, 2015; Byrne and Targher, 2015). These previous studies suggested that the gut microbiota may be responsible for the recovery of the tight junctions in the intestine and the insulin signaling pathway to improve liver steatosis and insulin resistance in NAFLD.

In previous experiments, rodents have always been fed RSV simultaneously with an high-fat diet (HFD) before the occurrence of NAFLD. In our study, we tested the therapeutic effects of RSV in an established NAFLD mouse model that imitates the clinical condition (Charytoniuk et al., 2017). In addition, many studies have shown that RSV can influence the gut microbiota in various diseases; however, the gut microbiota also changes with the progression of diseases. Considering this, we cultured the gut microbiota with RSV *in vitro* under an anaerobic environment to verify that RSV had a direct impact on the gut microbiota.

## Materials and Methods

### Diets and Animals

Seven-week-old male C57BL/6J mice were obtained from Shanghai SLAC Laboratory Animal Technology Co., Ltd. The mice were maintained in a specific pathogen-free (SPF) standard environment.

The mice were fed a normal chow diet (NCD) for 1 week to allow them to acclimate to the environment. The mice were randomly divided into three groups: the NC, HFD, and RSV groups. The NC group (*n* = 5) was fed a NCD. The HFD group (*n* = 6) and RSV group (*n* = 8) were both fed a 60% HFD (MD12033, Medicience, Jiangsu, China). All the mice had free access to food and water. In the first 8 weeks, the HFD and RSV groups were maintained under the same conditions to establish the NAFLD model. Thereafter, the RSV group underwent oral gavage with resveratrol (RSV, R5010; Sigma-Aldrich; 50 mg/kg body weight) resolved in sodium carboxymethyl cellulose (CMC, 419273; Sigma-Aldrich) daily for the last 4 weeks, and the HFD and NC groups received gavage with the same volume of CMC. 2 days before sacrifice. Fecal samples were collected from each mouse using a sterilized metabolism cage. The mice were fasted for 16 h to detect fasting blood glucose and then sacrificed, and blood, liver, colon, and fat samples were collected. The liver and fat were weighed, and part of the liver samples was placed in 4% paraformaldehyde for histological analysis. All the samples were immediately frozen in liquid nitrogen and stored at −80°C. The study was approved by The Institutional Animal Care and Use Committee of Fujian Medical University (Fujian, China).

High dose RSV experiment: The mice were randomly divided into three groups: the NC, HFD, and HRSV groups, each group had six mice. The HRSV group underwent oral gavage with RSV (100 mg/kg body weight) resolved in sodium carboxymethyl cellulose, other treatments are the same as above.

Blank control experiment: 8-week-old male C57BL/6J mice were randomly divided into two groups: the NC (gavage CMC *n* = 6) and Blank groups (without gavage any substance *n* = 6), both groups were treated for 4 weeks and the mice were sacrificed and the tissue were collected (Supplementary Figures 4A–F).

### Histological Analysis

Liver sections were submerged in 4% paraformaldehyde for 24 h, followed with a standard hematoxylin and eosin (H&E) staining procedure. Oil red O staining were performed in liver tissues frozen in OCT and stained in oil red O working solution (Servicebio technology, Wuhan, China). All slides were photographed using a Leica DM2000 light microscope (Leica Microsystems, Inc.; magnification, x100 or x200).

### Biochemical Analysis

Blood samples were isolated by centrifugation at 4°C, 3,000 rpm for 10 min. Fasting plasma glucose was detected using a blood glucose meter (Roche), and insulin levels were tested by ELISA kit (R&D). Homeostasis model assessment of insulin resistance (HOMA-IR) formula: [glucose levels (mmol/L) × insulin levels (mU/L)]/22.5. The concentration of total cholesterol (TC), triglyceride (TG), low-density lipoprotein cholesterol (LDL-C), and high-density lipoprotein cholesterol (HDL-C) in plasma were determined by cobas 8000 (Roche), liver TG were detected by commercial kits (Nanjing Jiancheng Bio, Nanjing, China), liver GSH and GSSG were measured by commercial kits (Beyotime, Shanghai, China).

### Western Blot

Protein were extracted from mice tissue in radioimmunoprecipitation assay (RIPA) buffer (Beyotime, Shanghai, China), proteins were separated by SDS-PAGE and were visualized using ECL system (Bio-Rad, Hercules, CA, United States). Primary antibodies used in this study were as follows: Occludin Polyclonal Antibody (71–1,500) and ZO-1 Polyclonal Antibody (71–1,500) were purchased from Invitrogen (Carlsbad, CA, United States). IRS-1 (D23G12) Rabbit mAb (3407), Phospho-IRS-1 (Ser307) Antibody (2381), mTOR (7C10) Rabbit mAb (2983), Phospho-mTOR (Ser2448; D9C2) XP Rabbit mAb (5536) were purchased from Cell Signaling Technology (Danvers, MA, United States), Monoclonal Anti-β-Actin antibody were purchased from Sigma-Aldrich (St. Louis, MO, United States).

### Quantitative Real-Time PCR

Total RNA was isolated from mouse tissues using the Trizol reagent (Invitrogen, Carlsbad, CA, United States), cDNA synthesis was conducted with a cDNA synthesis kit (Thermo Scientific, Waltham, MA, United States). SYBR Green (Roche, Basel, Switzerland) was used in qRT-PCR by a 7,500 Real-Time PCR instrument (Applied Biosystems). PCR amplification used the following conditions: 95°C for 10 min, 95°C for 15 s (denature), and 60°C for 1 min (anneal/extend) for 40 cycles, 95°C for 15 s, and 60°C for 1 min and then 95°C for 15 s (Mel curve). Primers were listed in the (Supplementary Table 1).

### Gut Microbiota Culture *in vitro*

Our method was similar to those employed in previous studies (Gross et al., 2010; Parkar et al., 2013). We collected 2 *g* of mouse feces and homogenized the specimen in 10 ml Gifu anaerobic medium (GAM; Hopebio, Qingdao, China). Then, the samples were centrifuged at 600 rpm for 5 min, the supernatant was collected, and 1 ml of the supernatant was added to 49 ml of sterile GAM to form a bacterial solution. Different reagents were supplied in the solution: 200 μl RSV solution (dissolved in ethanol, 200 mg/ml; RSV group, *n* = 5), 200 μl ethanol (control group, *n* = 5), and no reagents (empty group, *n* = 5). The bacterial solution was homogenized under hypoxic conditions. Subsequently, all the solutions were placed in anaerobic culture bags (Hopebio, Qingdao, China), shaken at 180 rpm and 37°C, and cultured for 48 h. Finally, the solutions were centrifuged at 6,000 rpm for 15 min to collect the precipitate and then stored at −80°C until sequencing. All the liquids were placed in hypoxic conditions for 24 h before the experiment.

### Fecal DNA Extraction

DNA extraction was performed using an E.Z.N.A. Soil DNA Kit (Omega, United States). The samples were dispersed by vibrating with glass beads in the sample tube to improve the lysis efficiency. To ensure that the amounts of high-quality genomic DNA were adequate, the concentration of bacterial DNA was measured using a Qubit 2.0 (Life, United States).

### 16S rRNA Gene Sequencing

PCR was initiated immediately after the genomic DNA was extracted. Our target was the V3–V4 region of the bacterial 16S ribosomal RNA gene (rRNA). The region was amplified using KAPA HiFi Hot Start Ready Mix (2×; TaKaRa Bio Inc., Japan) with barcode primers (PAGE purified): forward primer (CCTACGGGNGGCWGCAG) and reverse primer (GACTACHVGGGTATCTAATCC). The amplicon product was purified with AMPure XP beads and quantified in a Qubit^®^ 2.0 Green double-stranded DNA assay. The amplicons from each reaction mixture were pooled in equimolar concentrations based on their quantification results. Sequencing was conducted using the Illumina MiSeq system (Illumina MiSeq, United States) following the manufacturer’s instructions.

### Microbial 16S rRNA Gene Sequence and Metagenomic Analyses

Operational taxonomic units (OTUs) were picked with a 97% similarity threshold with Usearch software. α-Diversity was calculated with Mothur based on the selected OTUs. RDP Classifier (V.2.12) was used to annotate these sequences taxonomically (Wang et al., 2007), and β-diversity was estimated according to the Bray–Curtis distance and visualized via principal coordinate analysis (PCoA). The differences in the abundance of bacteria between samples were analyzed with STAMP (V.2.1.3). The LEfSe test was used to distinguish representative differences among the groups in LEfSe software (V.1.1.0). To investigate the function of the microbiota, we utilized PICRUSt (Phylogenetic Investigation of Communities by Reconstruction of Unobserved States) to predict the functional gene composition of 16S rRNA sequences by comparison with the existing sequenced microbial genome (Langille et al., 2013). Then, the enrichment of the predicted functional genes in KEGG (Kyoto Encyclopedia of Genes and Genomes) level 3 pathways was analyzed.

### Statistical Analyses

All statistical analyses were performed using GraphPad Prism software (version 8.0; GraphPad Software, SD, United States) and R packages (V.3.6.3). Data are shown as the mean ± SEM. Normally distributed data were tested by one-way ANOVA followed by Tukey’s *post hoc* test or Student’s *t*-test. Other types of data were tested with the non-parametric Mann–Whitney test (Kruskal–Wallis test for multiple groups). ANOSIM was used to analyze the similarities of the microbiota. Differences between the taxa were evaluated by Welch’s test followed by false discovery rate (FDR) correction. Spearman’s rank test was performed for correlation analysis. Multiple comparisons were adjusted according to the Benjamini and Hochberg FDR. The *P* value thresholds were as follows: ^∗^*p* < 0.05; ^∗∗^*p* < 0.01; ^∗∗∗^*p* < 0.001; and ^****^p < 0.0001.

## Results

### RSV Ameliorated HFD-Induced Fatty Liver and Metabolic Disorders in Mice

**FIGURE 1:**
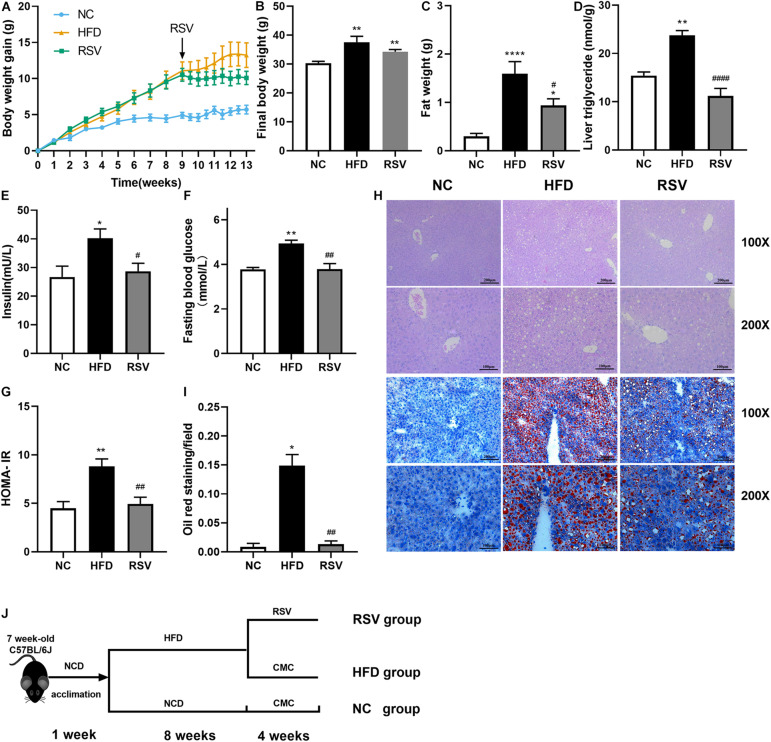
RSV had therapeutic effects on HFD-induced fatty liver and metabolic parameters in mice. **(A)** Weight gain curve. **(B)** Final body weight. **(C)** Fat weight. **(D)** Liver triglycerides. **(E–G)** Glucose homeostasis indicators: serum insulin, fasting blood glucose, and calculated HOMA-IR. **(H)** H&E staining and oil red O staining of mouse livers. **(I)** Quantification of the red area/total area by oil red O staining. **(J)** Graphical protocol of animal experiment. Data are shown as the mean ± SEM, variables were analyzed using Tukey’s test or the Mann–Whitney test. *p < 0.05; **p < 0.01; and ****p < 0.0001 vs NC group, ^#^*p* < 0.05; ^##^*p* < 0.01; and ^####^*p* < 0.0001 vs HFD group.

A NAFLD mouse model was established after 8 weeks of HFD feeding, followed by treatment with RSV (50 mg/kg, RSV group or 100 mg/kg, and HRSV group) for 4 weeks to verify its therapeutic effect. The body weight of the mice was significantly increased post HFD feeding, and the curve of all the RSV treatment group was separated from that of the HFD group over the last 4 weeks (Figure 1A and Supplementary Figure 1A). At the end of the experiment, we found that compared with the HFD group, the RSV group exhibited a decreasing trend in indicators such as final body weight (Figure 1B) and serum ALT, AST, TG, CHOL, and LDL-C levels, but the differences were not statistically significant (Table 1). RSV treatment had no effect on liver weight or serum HDL-C (Table 1). However, the HRSV group significantly decreased the final body weight (Supplementary Figure 1B). In both different dosage groups, RSV attenuated the increase in adipose tissue weight induced by HFD (Figure 1C and Supplementary Figure 1C) and reversed the HFD-induced high liver TG level (Figure 1D and Supplementary Figure 1D). We also found that RSV improved glucose homeostasis by reducing insulin levels, fasting blood glucose, and HOMA-IR (Figures 1E–G and Supplementary Figures 1E–G). At the same time, we identified the pathological state of the liver by using H&E staining and oil red O staining. H&E staining showed less ballooning degeneration in the RSV group than in the HFD group, and oil red O staining revealed an obvious shrinkage of the red staining area in the RSV-treated mice (Figure 1H). then we quantified the oil red O staining area (Figure 1I). The same changes were also observed in the HRSV group (Supplementary Figures 1H,I). Finally, we summarized our animal experiments by a graphical protocol (Figure 1J). The results demonstrated that RSV plays a role in improving hepatic steatosis.

**TABLE 1:** Metabolic variables in the three groups at the end of RSV treatment.

Parameters	NC	HFD	RSV
Liver weight (g)	1.12 ± 0.03	1.19 ± 0.1	1.25 ± 0.02
Serum parameters			
ALT (U/L)	26.8 ± 2.48	39.67 ± 7.25	38.38 ± 9.86
AST (U/L)	135 ± 24.1	186.83 ± 13.52	156.88 ± 20.83
TG (mmol/L)	0.52 ± 0.03	0.63 ± 0.05	0.48 ± 0.03
CHOL (mmol/L)	2.63 ± 0.12	3.69 ± 0.24	3.4 ± 0.27
HDL-C (mmol/L)	1.9 ± 0.08	2.24 ± 0.2	2.35 ± 0.07
LDL-C (mmol/L)	0.37 ± 0.04	0.92 ± 0.17^∗^	0.68 ± 0.14

*The data are shown as the mean ± SEM. Statistical differences were measured using Tukey’s test or the non-parametric Mann–Whitney test. Symbols indicating significant differences compared with the NC group: ^∗^p < 0.05.*

### RSV Remodeled the Composition and Abundance of the Gut Microbiota in HFD-Fed Mice

**FIGURE 2:**
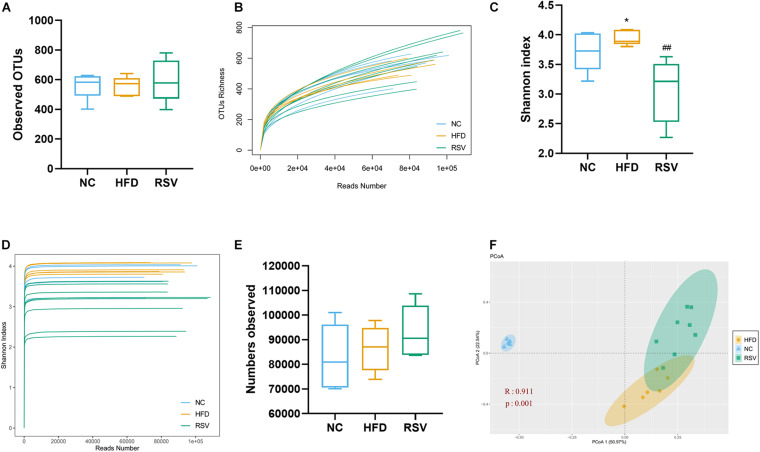
RSV modulated the diversity of the gut microbiota in NAFLD mice. Representative alpha diversity analysis factors: **(A)** OTUs richness. **(B)** Rarefaction curves at the OTU level, **(C)** Shannon index. **(D)** Shannon rarefaction curves. **(E)** Observed microbiota sequence numbers. Representative β-diversity analysis factor: **(F)** Principle coordinate analysis (PCoA) of the fecal microbiota at the OTU level. Data are shown as the mean ± SEM, variables were analyzed using Tukey’s test, Similarities were analyzed by ANOSIM. *p < 0.05 vs NC group, ^##^*p* < 0.01 vs HFD group.

To assess the changes in the gut microorganisms caused by RSV treatment in an established NAFLD model, we sequenced the V3-V4 regions of the 16S rRNA gene of the gut flora using the Illumina MiSeq platform. The sequencing results are presented as OTUs. HFD feeding had no effect on the observed OTUs, and we found no difference in the overall OTU numbers among all the groups (Figures 2A,B), indicating that they presented the same microbiome richness. Additionally, the sequencing depth covered most of the diversity according to the OTU richness rarefaction curves. However, our data showed that the Shannon index was increased in the HFD-fed mice compared with those fed a normal diet. The Shannon index is an important alpha diversity indicator and implied that HFD feeding could increase the diversity of the gut flora, whereas it was decreased by RSV treatment (Figure 2C). The curves indicated that our data covered most of the Shannon diversity (Figure 2D). We found no significant difference in the microbiota sequence numbers among all the groups (Figure 2E), indicating that all the groups had the similar amount of microbiota. In accordance with it, in the high dose RSV experiment, we found no significant difference in the number of bacteria (Supplementary Figure 2A), however, the Shannon index was decreased by high dose RSV treatment compared with the HFD group (Supplementary Figure 2B). β diversity was evaluated by Bray-Curtis dissimilarity-based PCoA (Figure 2F and Supplementary Figure 2C), which revealed distinctive clustering of the gut flora composition in each group, and the difference was significant. The RSV and HRSV group exhibited microbiota composition that clustered separately from that of the HFD group, indicating that RSV altered the gut microbiota of NAFLD mice.

**FIGURE 3:**
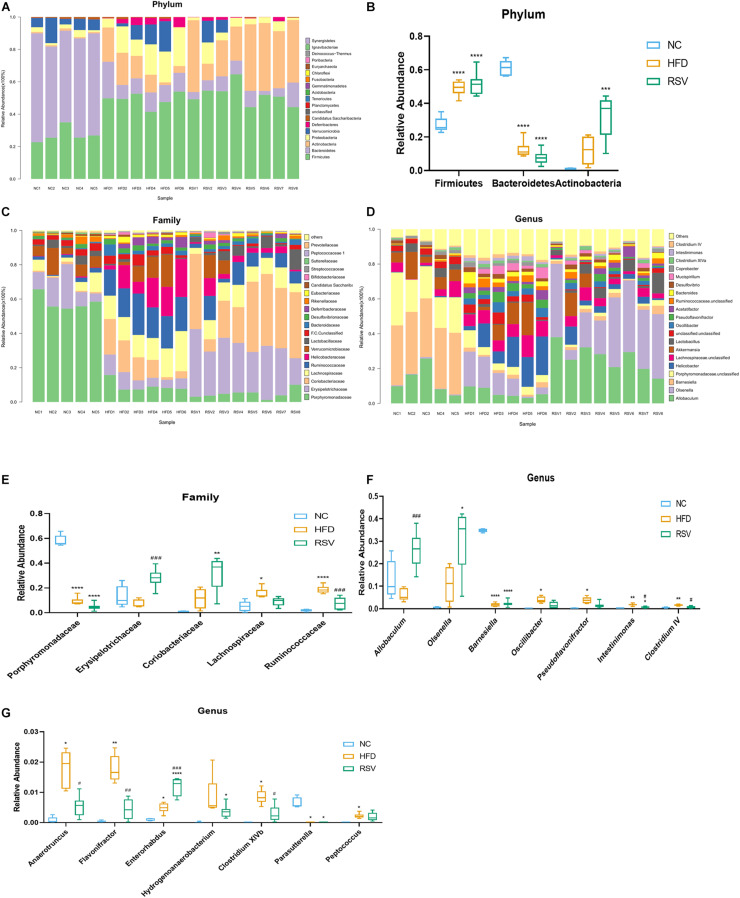
RSV caused significant changes in the gut microbiota at the phylum, family, and genus levels. **(A)** Changes in the composition of the gut microbiota in different groups at the phylum level; stacked bar charts represent the relative abundance of major taxa. **(B)** The relative abundance of bacteria differed in the three groups at the phylum level. **(C)** Composition of the gut microbiota at the family level. **(D)** Composition of the gut microbiota at the genus level. **(E)** The relative abundance of bacteria differed in the three groups at the family level. **(F,G)** The relative abundance of bacteria differed in the three groups at the genus level. Data are shown as the mean ± SEM. Variables were analyzed using Welch’s test followed by FDR correction. *p < 0.05; **p < 0.01; ***p < 0.001; and ****p < 0.0001 vs NC group, ^#^*p* < 0.05; ^##^*p* < 0.01; ^###^*p* < 0.001; and ^####^*p* < 0.0001 vs HFD group.

To determine the specific changes in the gut microbiota of all groups, we used a bar plot to show the composition of the predominant flora in each group and observed significant changes in the microbiota at different levels. In the low dose RSV experiment, at the phylum level, the predominant bacteria were *Firmicutes, Bacteroidetes*, and *Actinobacteria* (Figure 3A). *Firmicutes* were significantly increased and *Bacteroidetes* were significantly decreased in both the HFD and RSV groups compared with the NC group, and RSV increased the relative abundance of *Actinobacteria* compared with the NC group (Figure 3B). At the family level, compared with the NC group, *Porphyromonadaceae* was decreased in both other groups, and *Lachnospiraceae* and *Ruminococcaceae* were elevated in the HFD group. However, RSV tended to promote *Erysipelotrichaceae* growth and suppress *Ruminococcaceae* growth compared with that in the HFD group (Figures 3C,E). At the genus level, more changes could be observed. Compared with the NC group, decreases in *Barnesiella* and *Parasutterella* and increases *Intestinimonas* and *Enterorhabdus* were observed in both the RSV and HFD groups. Additionally, HFD increased the relative abundance of *Oscillibacter*, *Clostridium IV*, *Pseudoflavonifractor*, *Anaerotruncus*, *Clostridium XlVb*, and *Peptococcus*, and RSV increased that of *Olsenella* and *Hydrogenoanaerobacterium*. Compared with the HFD group, RSV had an increasing effect on *Allobaculum* and *Enterorhabdus* and a decreasing effect *Intestinimonas*, *Clostridium IV*, *Anaerotruncus*, *Flavonifractor*, and *Clostridium XlVb* (Figures 3D,F,G). In the high dose RSV experiment, at the phylum level, *Firmicutes* were significantly increased and *Bacteroidetes* were significantly decreased in both the HFD and HRSV groups compared with the NC group, and high dose RSV further increased the *Firmicutes* compared with HFD group (Supplementary Figures 3A,C). At the family level, compared with the NC group, *Porphyromonadaceae* was decreased and *Erysipelotrichaceae* was increased in both other groups (Supplementary Figures 3B,E). At the genus level, more changes could be observed. Compared with the NC group, decreases in *Barnesiella* and increases in *Allobaculum* were observed in both the HRSV and HFD groups. Additionally, HRSV increased that of *Olsenella* compared with the NC group, and can further promote *Allobaculum* compared with the HFD group (Supplementary Figures 3D,F).

### Metabolic Pathways Were Changed After RSV Treatment in HFD Mice

**FIGURE 4:**
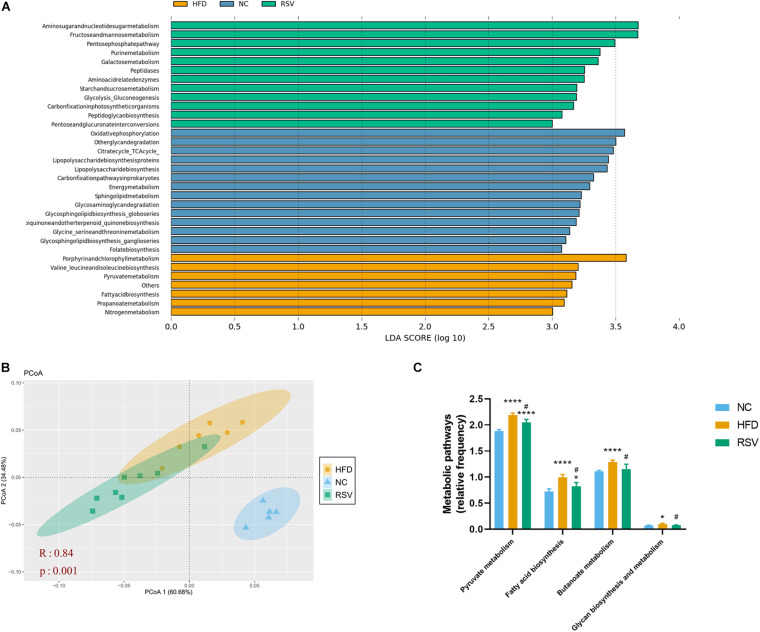
RSV changed the function of the gut microbiota in HFD-fed mice. **(A)** The linear discriminant analysis (LDA) effect size (LEfSe) showed the relevant KEGG pathways predicted by PICRUSt (LDA score > 3.0 and significance of *P* < 0.05 analyzed by the Wilcoxon signed-rank test). **(B)** Principle coordinate analysis (PCoA) showed the Bray–Curtis distance of the predicted KEGG pathways in the three groups. **(C)** Glucose and lipid metabolic pathways that were altered between groups. Data are shown as the mean ± SEM, the Kruskal–Wallis and Wilcoxon tests were performed in LEfSe, ANOSIM was used for similarities in PCoA. *p < 0.05; and ****p < 0.0001 vs NC group, ^#^*p* < 0.05 vs HFD group.

PICRUSt is a tool for predicting microbial community function based on 16S rRNA gene sequencing. We compared our data with existing 16S rDNA sequencing databases to analyze the functional differences between different samples and groups. The obtained prediction results were used to enrich gene families by KEGG analysis. NAFLD was highly relevant to various metabolic pathways, so we used the linear discriminant analysis (LDA) effect size (LEfSe) to analyze the differences in metabolic pathways between the three groups (Figure 4A). As a result, changes in amino acid, carbohydrate, energy, glycan, and lipid metabolism were observed. Most carbohydrate metabolic pathways, including fructose and mannose metabolism, glycolysis gluconeogenesis, galactose metabolism and the pentose phosphate pathway, were enriched in the RSV group; most of the glycan biosynthesis pathways were enriched in the NC group; and lipid metabolic pathways such as fatty acid biosynthesis were enriched in the HFD group. Bray–Curtis-based PCoA also revealed a significant difference in gene function in the three groups (Figure 4B). Subsequently, we listed some glucose and lipid metabolism modules, including pyruvate metabolism, fatty acid biosynthesis, butanoate metabolism, glycan biosynthesis and metabolism, that were significantly increased in the HFD groups and decreased in the RSV group (Figure 4C), indicating that RSV may exert effects through these pathways.

### RSV Restored Gut Tight Junctions and Ameliorated Liver Inflammation

**FIGURE 5:**
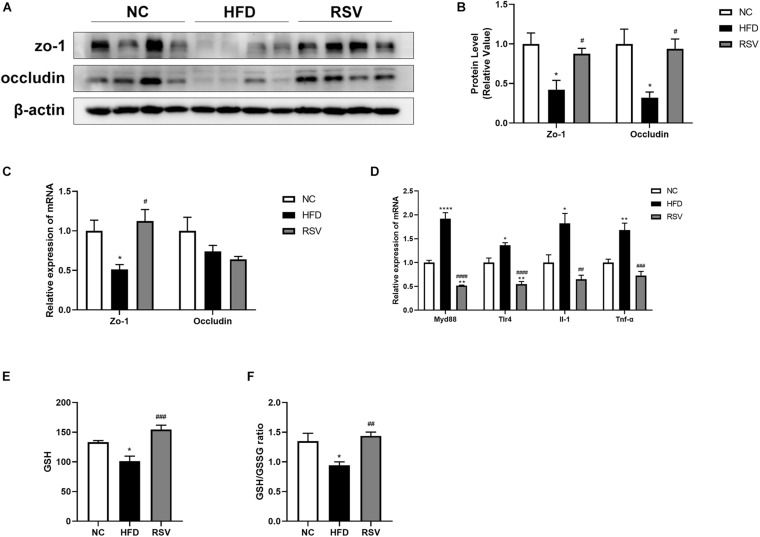
RSV restored the loss of tight junctions caused by HFD in the gut and ameliorated liver inflammation and oxidative stress. **(A)** Protein levels of the tight junction factors (zo-1, occludin) in the colon among different groups. **(B)** Statistical analysis of protein levels. **(C)** Relative mRNA expression of zo-1 and occludin in the mouse colon. **(D)** Relative mRNA expression of inflammatory factors (MyD88, TLR-4, IL-1, and TNF-α) in the liver. **(E)** Total liver GSH level. **(F)** Total liver GSH/GSSG ratio. Data are shown as the mean ± SEM, variables were analyzed using Tukey’s test, *p < 0.05; **p < 0.01; and ****p < 0.0001 vs NC group, ^#^*p* < 0.05; ^##^*p* < 0.01; ^###^*p* < 0.001; and ^####^*p* < 0.0001 vs HFD group.

Tight junctions in the intestinal epithelium are an important barrier for preventing harmful substances from entering the gut-liver axis. To determine whether tight junctions experienced changes during the development of the disease, we measured the protein and mRNA expression levels of the tight junction proteins zo-1 and occludin in the colon (Figures 5A–C). We found that in the HFD group, the protein expression levels of zo-1 and occludin decreased but could be restored after RSV treatment. Additionally, the gene expression of zo-1 decreased in the HFD group and was upregulated in the RSV group. There was no change in the gene expression of occludin. Subsequently, we examined the signaling pathways related to the immune response in the liver, the gene expression of inflammatory factors (Figure 5D), and the level of oxidative stress in the liver (Figures 5E,F) and found that immune signaling molecules such as TLR4 and MyD88, and inflammatory factors such as IL-1 and TNF-α were increased in the HFD group and that these changes were reversed in the RSV group. The liver oxidative stress indicators GSH and GSH/GSSG decreased in the HFD group and increased in the RSV group. The above results suggest that RSV can repair the damage to tight junctions in the intestine caused by HFD and simultaneously reduce the liver immune response, inflammatory factor levels and the oxidative stress level.

### RSV Repaired the Insulin Signaling Pathway and Altered Fatty Acid Metabolism-Related Gene Expression

**FIGURE 6:**
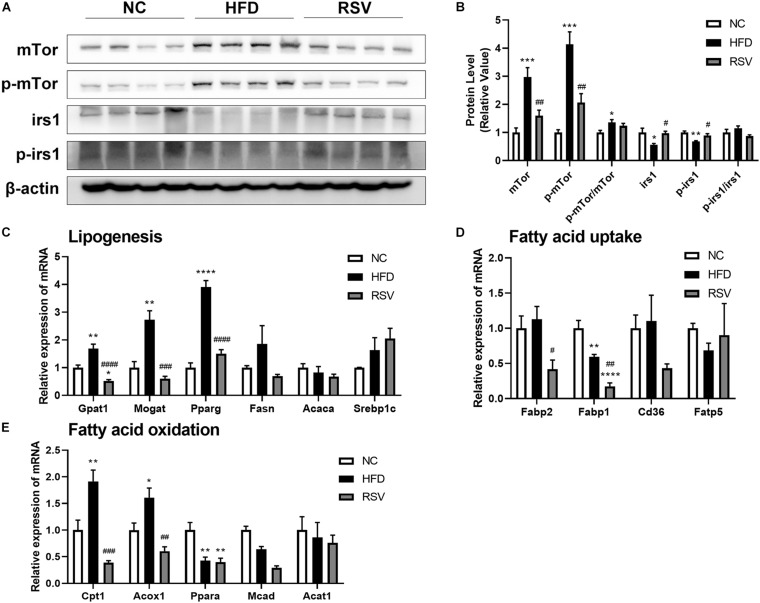
Effects of resveratrol on the insulin signaling pathway and fatty acid-related genes in fatty liver mice. **(A)** Western blotting was used to detect the protein levels of key molecules in the insulin signaling pathway (irs1, p-irs1. mTor, and p-mTor) in the liver among the different groups. **(B)** Statistical analysis of the protein levels. The relative expressions of genes related to **(C)** lipogenesis, **(D)** fatty acid uptake, and **(E)** fatty acid oxidation were assessed by qRT-PCR. Data are shown as the mean ± SEM, variables were analyzed using Tukey’s test, *p < 0.05; **p < 0.01; ***p < 0.001; and ****p < 0.0001 vs NC group, ^#^*p* < 0.05; ^##^*p* < 0.01; ^###^*p* < 0.001; and ^####^*p* < 0.0001 vs HFD group.

The insulin signaling pathway plays an important role in fatty acid metabolism. Our study indicated that RSV is beneficial for improving insulin resistance, so we tested the key molecules of the insulin signaling pathway and genes related to lipogenesis, fatty acid oxidation, and fatty acid uptake. We found that both irs1 and p-irs1 were decreased in the HFD group and recovered in the RSV group, while mTor and p-mTor both increased in the HFD group and decreased after RSV treatment. However, we did not observe significant changes in p-irs1/irs1 and p-mTor/mTor (Figures 6A,B), proving that RSV can repair the damaged insulin signaling pathway, mainly by affecting the overall protein level and phosphorylated protein level of molecules in the pathway but not by affecting the ratio of phosphorylation. Among fatty acid metabolism genes, we found that some lipogenesis-related genes, such as Gpat1, Mogat, and Pparg, were inhibited by RSV treatment (Figure 6C). Although we did not find that HFD caused increased expression of liver fatty acid uptake-related genes in our experiments, RSV did suppress genes such as Fabp2 and Fabp1 (Figure 6D). We also noted that fatty acid oxidation-related genes (Cpt1, Acox1) showed increases in expression in the HFD group and that these increases were abolished after the administration of RSV (Figure 6E). The above results indicated that RSV can restore the expression of genes related to lipid metabolism to normal levels.

### The Gut Microbiota Was Correlated With Metabolic Parameters

**FIGURE 7:**
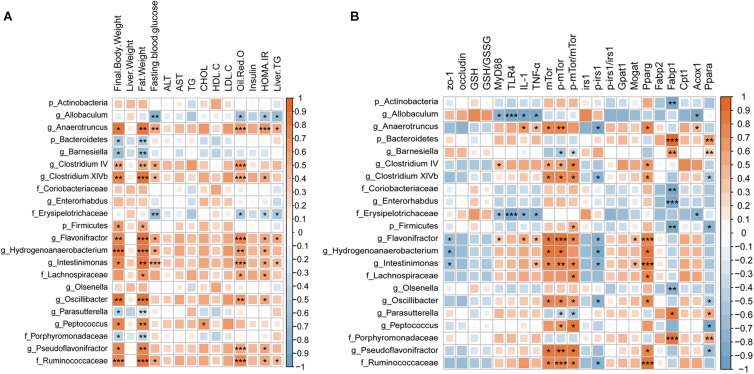
Spearman’s correlation coefficient heat map showing the association between the changes in the gut microbiota and **(A)** metabolic biomarkers and **(B)** molecules, followed by FDR correction. Red represents a positive correlation; blue represents a negative correlation. Deeper color indicates an increased correlation coefficient. Significance threshold: *p < 0.05; **p < 0.01; and ***p < 0.001.

We performed Spearman’s correlation analysis followed by FDR correction to explore the relationship between the altered gut microbiota and metabolic indicators (Figure 7A). We found that the gut microbiota was mainly related to body weight, fat weight, fasting blood glucose, HOMA-IR, oil red O staining, and liver TG. 12 taxa (*Firmicutes*, *Lachnospiraceae*, *Pseudoflavonifractor*, *Intestinimonas*, *Ruminococcaceae*, *Flavonifractor*, *Clostridium XlVb*, *Peptococcus*, *Oscillibacter*, *Anaerotruncus*, and *Hydrogenoanaerobacterium*) shown in the graph were positively related to these indicators, while six taxa (*Barnesiella*, *Bacteroidetes*, *Porphyromonadaceae*, *Parasutterella*, and *Allobaculum*) were negatively related to them. Furthermore, we calculated the correlation coefficient of the gut microbiota and previously detected signaling molecules (Figure 7B) and found that most of the molecules exhibiting changes after RSV treatment presented a significant correlation with the gut microbiota. It is worth mentioning that mTor, p-mTor, p-irs1, and pparg were related to similar components of the microbiota (*Hydrogenoanaerobacterium*, *Anaerotruncus*, *Flavonifractor*, *Oscillibacter*, *Ruminococcaceae*, *Intestinimonas*, and *Clostridium XlVb*); mTor, p-mTor, and pparg were positively related to these groups, while p-irs1 was negatively related to them. The above data showed that many metabolic indicators and molecules that were recovered by RSV treatment were related to the gut microbiota, suggesting that the gut microbiota is an important factor in the anti-NAFLD effect of RSV.

### RSV Changed the Composition of the Gut Microbiota *in vitro*

**FIGURE 8:**
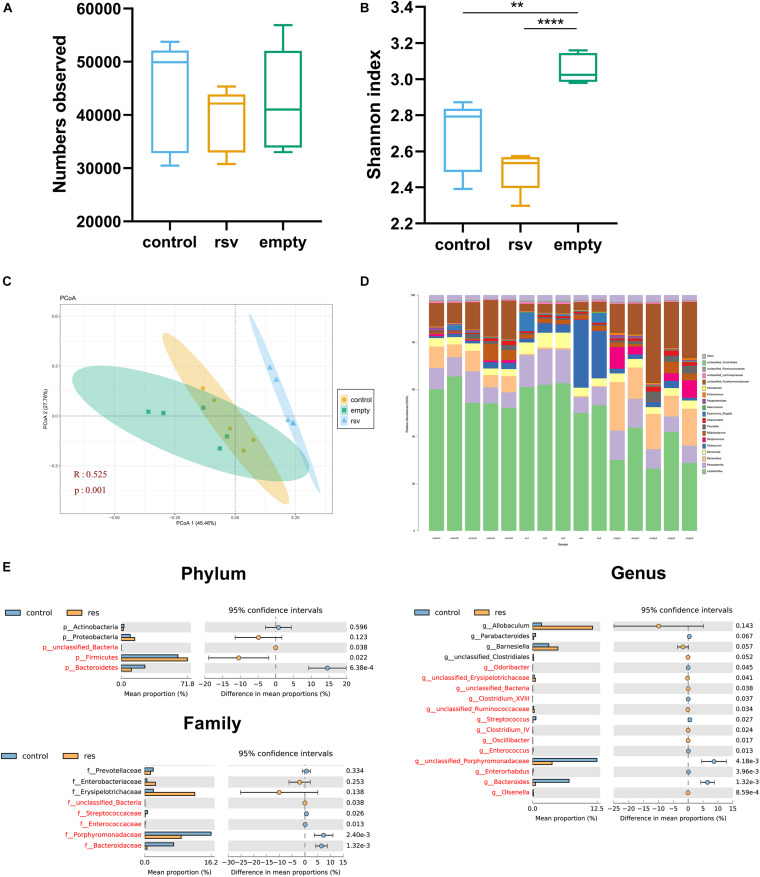
RSV changed the composition of the gut microbiota *in vitro*. Representative alpha diversity analysis factors: **(A)** Observed sequence numbers. **(B)** Shannon index. Representative β-diversity analysis factor: **(C)** Principle coordinate analysis (PCoA) of the microbiome at the OTU level. **(D)** Changes in the composition of the microbiome at the genus level; stacked bar charts represent the relative abundance of taxa. **(E)** The relative abundance of the microbiome differed between the control and RSV groups at the phylum, families, and genus levels. Data are shown as the mean ± SEM. The α- diversity were measured by Tukey’s test; β-diversity were measured by ANOSIM; differences between taxa were measured by Welch’s test followed by FDR correction, **p < 0.01; and ****p < 0.0001.

To verify whether RSV had the ability to directly change the gut microbiota, we cultured the gut microbiota in GAM and then fermented the microbiota with RSV dissolved in ethanol (RSV group), with an equal dose of ethanol (control group) or with no additional treatment (empty group). The fermented samples were collected at 48 h, after which 16S rRNA sequencing was performed. The results showed that the sequence numbers had no significance in all the groups (Figure 8A) and that the Shannon indexes of control and rsv groups were lower than those of the empty group (Figure 8B). At the same time, we measured the β diversity by PCoA and found that RSV significantly changed the community composition during fecal fermentation (Figure 8C). Subsequently, we analyzed the changes in taxa at different levels between the control and RSV groups (Figures 8D,E). At the phylum level, RSV increased the relative abundance of *Firmicutes* and decreased that of *Bacteroidetes*. At the family level, we observed decreasing abundance of *Porphyromonadaceae, Bacterioidaceae, Enterococcaceae, Streptococcaceae*. At the genus level, we found increases in *Olsenella*, *Oscillibacter*, and *Clostridium_IV* and decreases in *Bacteroides*, *Streptococcus*, and *Enterorhabdus*. The above results confirmed that RSV can independently change the composition of the gut microbiota *in vitro*.

## Discussion

Currently, there is no reliable medical treatment for non-alcoholic fatty liver (Sumida and Yoneda, 2018). In our study, we verified the therapeutic effect of RSV on NAFLD mice and explored the underlying mechanism. The results showed that RSV can significantly improve liver steatosis and insulin resistance in mice. We also observed a downward trend in body weight and blood indicators after RSV treatment, suggesting that RSV exerted a specific effect on improving NAFLD.

The gut microbiota is a new emerging target of RSV. We detected the gut microbiota of the three groups by 16S rRNA sequencing and observed significant changes. In terms of α-diversity, we noted that the Shannon index was increased in the HFD group and decreased in both low and high dose RSV group. However, some studies have indicated that patients with NAFLD exhibit lower α-diversity (Murphy et al., 2010; Wilmanski et al., 2019), which could be caused by the complicating lifestyle factors of patients; in addition to consuming a HFD, they also consume high carbohydrate levels and exhibit a lack of exercise. Subsequently, we analyzed the specific differences in taxa among the gut microbiota and evaluated their correlation with metabolic indicators. Consistent with some previous studies, we found that HFD significantly increased *Firmicutes* and reduced *Bacteroidetes* at the phylum level, but low dose RSV treatment didn’t reverse the changes on these phyla (Yang et al., 2019), the high dose RSV further elevated the *Firmicutes* level. However, other studies have suggested that RSV can restore *Firmicutes* and *Bacteroidetes* (Zhao et al., 2017). These differences may be caused by the timing of the medication. In our study, mice were treated with RSV after NAFLD model is established to verify the therapeutic effect, other previous studies treated mice with RSV at the beginning of HFD feeding, and the duration of medication is different between the studies. In the low dose RSV experiment, RSV caused a significantly increase in the *Actinobacteria* phylum, along with the *Olsenella* and *Enterorhabdus* genera (*Coriobacteriaceae* family). *Olsenella* can produce SCFAs, which are related to the tight junctions of the intestinal barrier. One study showed that pectin can increase *Olsenella* and ameliorate fatty liver (Li et al., 2018). We found that most of the altered genera belonged to *Firmicutes*, but the correlation with metabolic indicators differed among genera. *Allobaculum* (*Erysipelotrichaceae* family) was negatively correlated with the oil red O staining area and fasting blood glucose. RSV could increase the proportion of *Allobaculum*. Previous studies have proven that increases in *Allobaculum* are beneficial to HFD mice (Raza et al., 2017; Li et al., 2019). *Allobaculum* shows increases when dietary fiber is taken and is negatively related to the concentration of leptin (Ravussin et al., 2012). In contrast, the *Lachnospiraceae* family (*Clostridium XlVb* genus) and *Ruminococcaceae* family (*Anaerotruncus*, *Clostridium IV*, *Flavonifractor*, *Intestinimonas*, and *Oscillibacter* genus) were all positively correlated with the severity of the disease, and RSV caused decreases in these genera. *Clostridium XlVb* is an anaerobic bacterium that was found to be reduced in mice receiving fruit extracts rich in polyphenols. It is related to the improvement of the obesity phenotype (Xu et al., 2019). Many experiments have shown that the *Ruminococcaceae* family is downregulated in NAFLD (Safari and Gérard, 2019; Chen et al., 2020), but in articles focused on severe stages of NAFLD, such as NASH and HCC, the *Ruminococcaceae* family has been reported to be upregulated in the disease group (Machado and Cortez-Pinto, 2016; Byrne and Targher, 2020), indicating that dynamic changes in the *Ruminococcaceae* family might occur in the process of disease development. The members of the *Anaerotruncus* and *Oscillibacter* genera are conditional pathogenic bacteria that in in high-fat and high-sugar diet-fed mice (Kong et al., 2019). Clostridium IV is positively correlated with a high-carbohydrate diet (Yamaguchi et al., 2016) and was found to be downregulated after bariatric surgery and probiotic treatment (Seganfredo et al., 2017). *Flavonifractor* has also shown a positive correlation with TG levels in some studies (Li et al., 2019). *Intestinimonas* is believed to increase in obese rats and is related to an increase in deoxycholic acid (Lin et al., 2019). In the high dose RSV study, we still observed the composition of microbiota is totally different in the three groups, however, the altered microbiota were not totally in consistent with the low dose group, the interaction between microbiota is very complex, we always seen various results in studies using different dosage and duration of RSV (Charytoniuk et al., 2017), meanwhile, the different batch of mice would also affect our result. However, we still notice some similar changes, the *Allocaculum* were increased in different dose group compared with the HFD group, and *Olsenella* genus were increased in different dose group compared with NC group, indicating these two genus might be an important target of RSV. Our data confirmed that RSV supplementation can indeed alter the gut microbiota of NAFLD mice. According to the correlation analysis, the altered gut microbiota is associated with the phenotype of NAFLD, and such alterations in the gut microbiota have been proven to cause beneficial changes in NAFLD in previous studies. Due to the limitation of sequencing depth, we can only distinguish the genera related to the disease, and more advanced sequencing is needed to identify the relevant functional species.

To further explore the underlying mechanisms, we tested tight junction proteins (zo-1 and occludin) in the colon and found that RSV can repair the damage to tight junctions caused by HFD. Recently, it has come to be generally believed that the “gut-liver axis” is a vital route whereby the gut microbiota affect liver metabolism. Previous studies have shown that changes in the composition and metabolites of the gut microbiota directly affect tight junctions (Lee et al., 2018). Our results also suggested that RSV modifies the intestinal immune response. It is generally believed that gut microbiota triggers innate and adaptive immunity and maintains the stability of the intestine (Hooper and Macpherson, 2010). In previous studies about Inflammatory Bowel Disease and Diabetic Nephropathy, the transplantation of fecal microbiota from RSV gavage mice directly affected the intestinal inflammation and gut barrier of the recipient mice (Alrafas et al., 2019; Cai et al., 2020), indicating the RSV might alter the intestinal inflammation by gut microbiota. We also observed reductions in liver inflammation and oxidative stress after RSV treatment. According to previous studies, the gut microbiota produces numerous immunogens, such as LPS, during metabolic processes. Damage to tight junctions causes excessive levels of LPS and other immunogens to enter the liver, stimulates the immune response, produces excessive inflammatory factors, and increases oxidative stress (Zhang et al., 2000; Kim et al., 2012). Another study demonstrated that HFD-induced damage to intestinal permeability leads to insulin resistance (Cani et al., 2007). Similar to a previous study, our experiments showed the recovery of fasting blood glucose and insulin levels, and the enrichment of glucose metabolic pathways was impaired after RSV treatment; therefore, we examined the insulin signaling pathway. The results showed that the effects on IRS1, p-IRS1, mTor and p-mTor resulting from HFD feeding can be repaired by RSV treatment. This may result from a decrease in liver inflammation because the inflammatory state of the liver can affect the insulin signaling pathway (Tilg and Moschen, 2008). In addition, we conducted correlation analysis and found that the tight junction and insulin signaling pathways were significantly related to the altered gut microbiota. In accordance with our results, previous studies have shown that RSV ameliorates insulin resistance in obese mice fed a high-fat and high-sugar diet; when the gut microbiota was transferred from RSV-treated mice to non-treated mice, the normalization of insulin resistance was observed in recipient mice, indicating that gut microbiota mediates the effects of RSV (Abbasi Oshaghi et al., 2017; Sung et al., 2017). Furthermore, we detected fatty acid metabolism genes and found that RSV reduced the expression of lipogenesis and fatty acid uptake genes, which can directly prevent steatosis. Previous studies had found that RSV can regulate fatty acid metabolism in the liver and adipose tissue (Alberdi et al., 2011; Andrade et al., 2014), moreover, the transplantation of feces microbiota from RSV gavage mice still had the ability to change the fatty acid metabolism in the recipient mice (Wang et al., 2020), indicating gut microbiota can alter the fatty acid metabolism independently. What’s more, we found that RSV can affect the insulin signaling pathway and the changes is associated with altered gut microbiota, previous findings showed that changes in the insulin signaling pathway can affect liver steatosis by altering the lipid metabolism process (Leavens and Birnbaum, 2011; Smith et al., 2020), suggesting that RSV may also reduce fatty acid metabolism genes by improving the insulin signaling pathway. The specific mechanisms need further studies. These results suggest that RSV may benefit NAFLD by repairing tight junctions and improving the insulin signaling pathway.

Although many studies have focused on the effects of RSV on the gut microbiota in various diseases (Chaplin et al., 2018), it is not clear whether the improvement of the disease causes changes in the gut microbiota or RSV directly triggers changes in the gut microbiota. Previous studies showed RSV can direct affect specific bacteria *in vitro* (Dos Santos et al., 2019), our experiment is the first to ferment RSV with a bacterial solution *in vitro* to observe the changes in the gut microbiota. The results showed that RSV can significantly change the composition of the gut microbiota *in vitro*. Although there are many differences in the *in vitro* and *in vivo* culture environments, we observed that some of the same changes, such as increases in the *Firmicutes* phylum were observed in the *in vitro* study and *in vivo* HRSV group, the low dose RSV group also have a trend to increase it. the *Allocaculum* genus were upregulated by both low dose and high dose studies, and the *in vitro* study also showed high amount of *Allocaculum* in the rsv group. *Olsenella* genus were increased in both three studies, but controversial results still existed. The contradictions may have resulted from the interaction between the gut microbiota and disease. As previous studies have reported, RSV can also ameliorate NAFLD in other ways, and the state of the disease may in turn influence the gut microbiota (Charytoniuk et al., 2017). The above results confirmed that RSV is an independent influencing factor of the gut microbiota.

At present, some clinical studies found that RSV had beneficial effects on various metabolic-related diseases. In type 2 diabetes researches, RSV was capable of decreasing blood glucose and improving insulin resistance (Brasnyo et al., 2011; Movahed et al., 2013). Meanwhile, RSV increased serum adiponectin and inhibited atherothrombotic signals in patients with coronary heart disease (Tome-Carneiro et al., 2013). Previous studies also found that RSV could lower cardiovascular risk by reducing cholesterol, systolic blood pressure, and diastolic blood pressure in obese people (Huang et al., 2016). As for the clinical studies of NAFLD, some results suggested that RSV was able to alleviate liver steatosis, downregulated the BMI and serum bilirubin (Faghihzadeh et al., 2015). However, there were still studies that didn’t observe significant beneficial effects. Considering that the efficiency of the drug can be influenced by gut microbiota (Wilson and Nicholson, 2017), the diversity of human gut microbiota might be an important factor (Lozupone et al., 2012). Exploring the effect of RSV on the gut microbiota can help it to become a therapeutic drug for NAFLD.

Although we found that RSV exerted an obvious therapeutic effect on NAFLD and changed the composition of the gut microbiota, to further explore the effect of the altered gut microbiota on NAFLD, fecal microbiota transplantation (FMT) experiments are needed. Our research group is currently conducting FMT-related experiments. Additionally, there are numerous different species belonging to each genus, and individual species often have different functions (Walker et al., 2014). Hence, we will also need more advanced sequencing methods, such as metagenomics, to study the relationship between species and NAFLD.

In conclusion, this article verified the potential of RSV as a therapeutic regimen for NAFLD. Moreover, RSV can change the gut microbiota, and the altered microbiota is significantly related to improved liver steatosis and insulin resistance. Our study provides new evidence regarding the potential use of RSV as an effective medical treatment for NAFLD.

## Data Availability Statement

The datasets presented in this study can be found in online repositories. The names of the repository/repositories and accession number(s) can be found below: https://figshare.com/, https://doi.org/10.6084/m9.figshare.13017218.v1; https://figshare.com/, https://doi.org/10.6084/m9.figshare.13017269.v1; https://figshare.com/, https://doi.org/10.6084/m9.figshare.13482726.v1; and https://figshare.com/, https://doi.org/10.6084/m9.figshare.13482666.v1.

## Ethics Statement

The animal study was reviewed and approved by Laboratory Aniamal Welfare and Ethic Committe of Fujian Medical University.

## Author Contributions

FD, RH, FC, and XW: conceptualization. ZC and YH: methodology. RH, XY, XH, and BZ: formal analysis. FD, DL, and YW: investigation. RH: resources. FD: data curation. FD and RH: writing—original draft preparation. FC and XW: writing—review and editing. All authors have read and agreed to the published version of the manuscript.

## Conflict of Interest

The authors declare that the research was conducted in the absence of any commercial or financial relationships that could be construed as a potential conflict of interest.

## Supplementary Material

Supplementary Figure 1 High dose RSV had the same therapeutic effects on HFD-induced fatty liver and metabolic parameters in mice.

Supplementary Figure 2 High dose RSV modulated the diversity of the gut microbiota in NAFLD mice.

Supplementary Figure 3 High dose RSV caused significant changes in the gut microbiota at the phylum, family, and genus levels.

Supplementary Figure 4 CMC had no effects on metabolic parameters in mice.

Supplementary Table 1 Primers used in the quantitative real-time PCR.
