# Supplementary material for: Resveratrol Improves Liver Steatosis and Insulin Resistance in Non-alcoholic Fatty Liver Disease in Association With the Gut Microbiota
Source: Front Microbiol. 2021 Feb 23;12:611323. doi: 10.3389/fmicb.2021.611323 (PMC7942199; doi:10.3389/fmicb.2021.611323)
Supplement: Supplementary Figure 1 — High dose RSV had the same therapeutic effects on HFD-induced fatty liver and metabolic parameters in mice. [file Data_Sheet_1.docx]

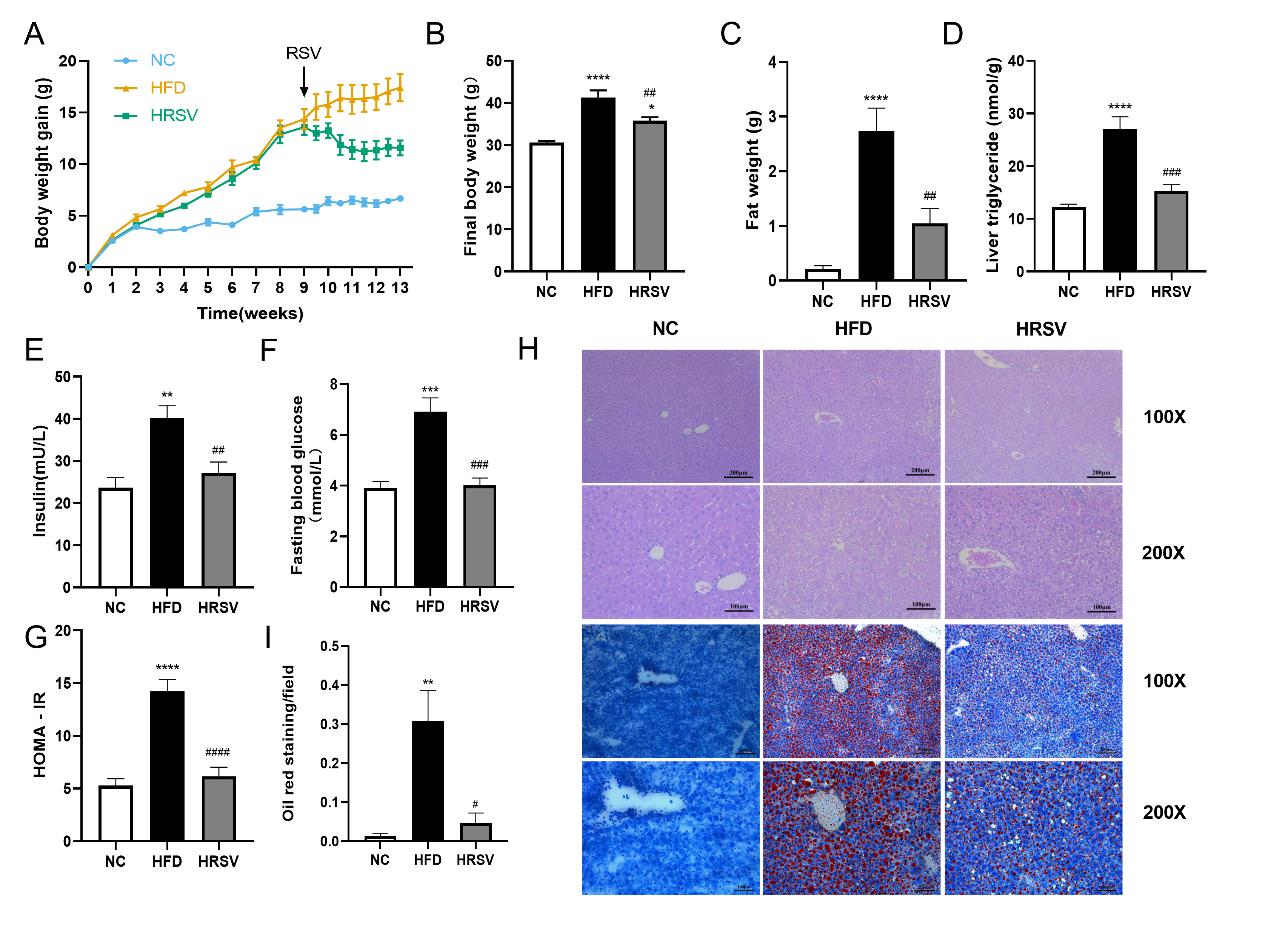


**Figure S1.** High dose RSV had the same therapeutic effects on HFD-induced fatty liver and metabolic parameters in mice. **(A)** Weight gain curve. **(B)** Final body weight. **(C)** Fat weight. **(D)** Liver triglycerides. **(E-G)** Glucose homeostasis indicators: serum insulin, fasting blood glucose and calculated HOMA-IR. **(H)** H&E staining and oil red O staining of mouse livers. **(I)** Quantification of the red area/total area by oil red O staining. Data are shown as the mean±SEM, variables were analysed using Tukey’s test or the Mann-Whitney test. *, p<0.05; **, p<0.01; ***, p<0.001; ****, p<0.0001 vs NC group, #, p<0.05; ##, p<0.01; ###, p<0.001; ####, p<0.0001 vs HFD group


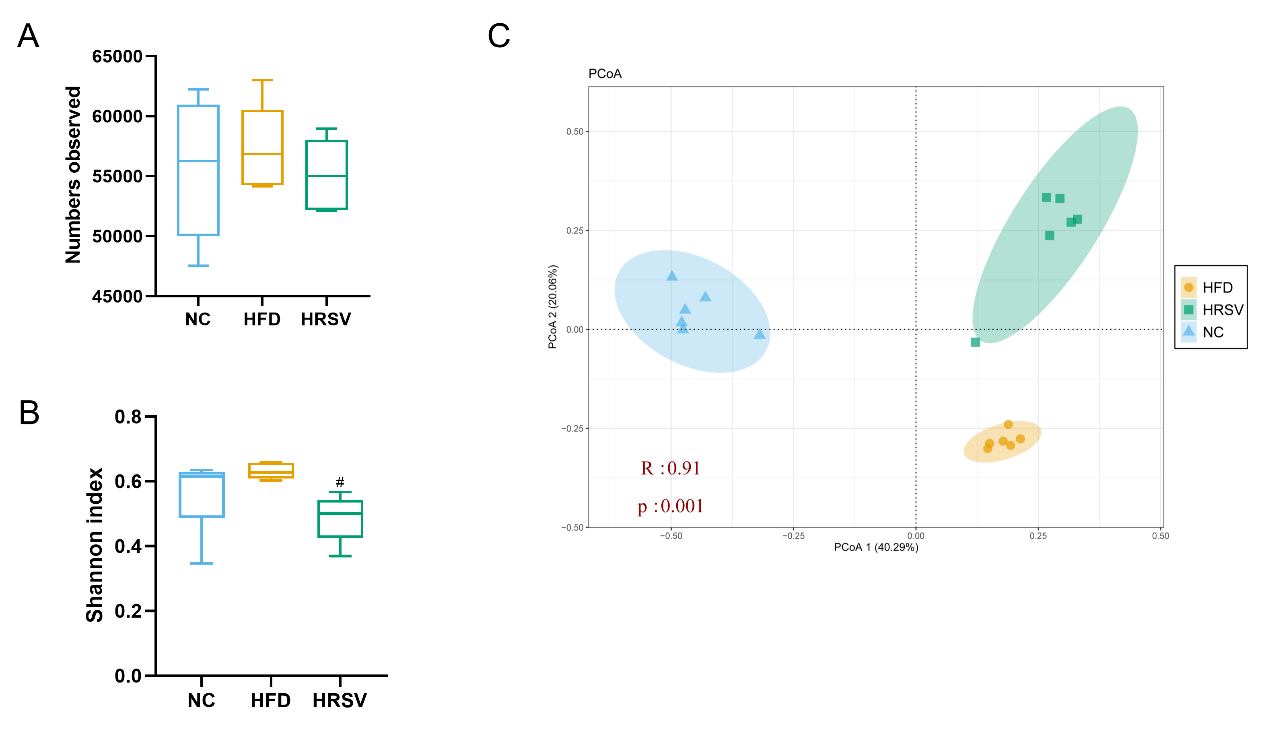


**Figure S2.** High dose RSV modulated the diversity of the gut microbiota in NAFLD mice. **(A)** Observed sequence numbers. **(B)** Shannon index. **(C)** Principle coordinate analysis (PCoA) of the faecal microbiota at the OTU level. Data are shown as the mean±SEM, variables were analysed using Tukey’s test, Similarities were analysed by ANOSIM. #, p<0.05 vs HFD group.


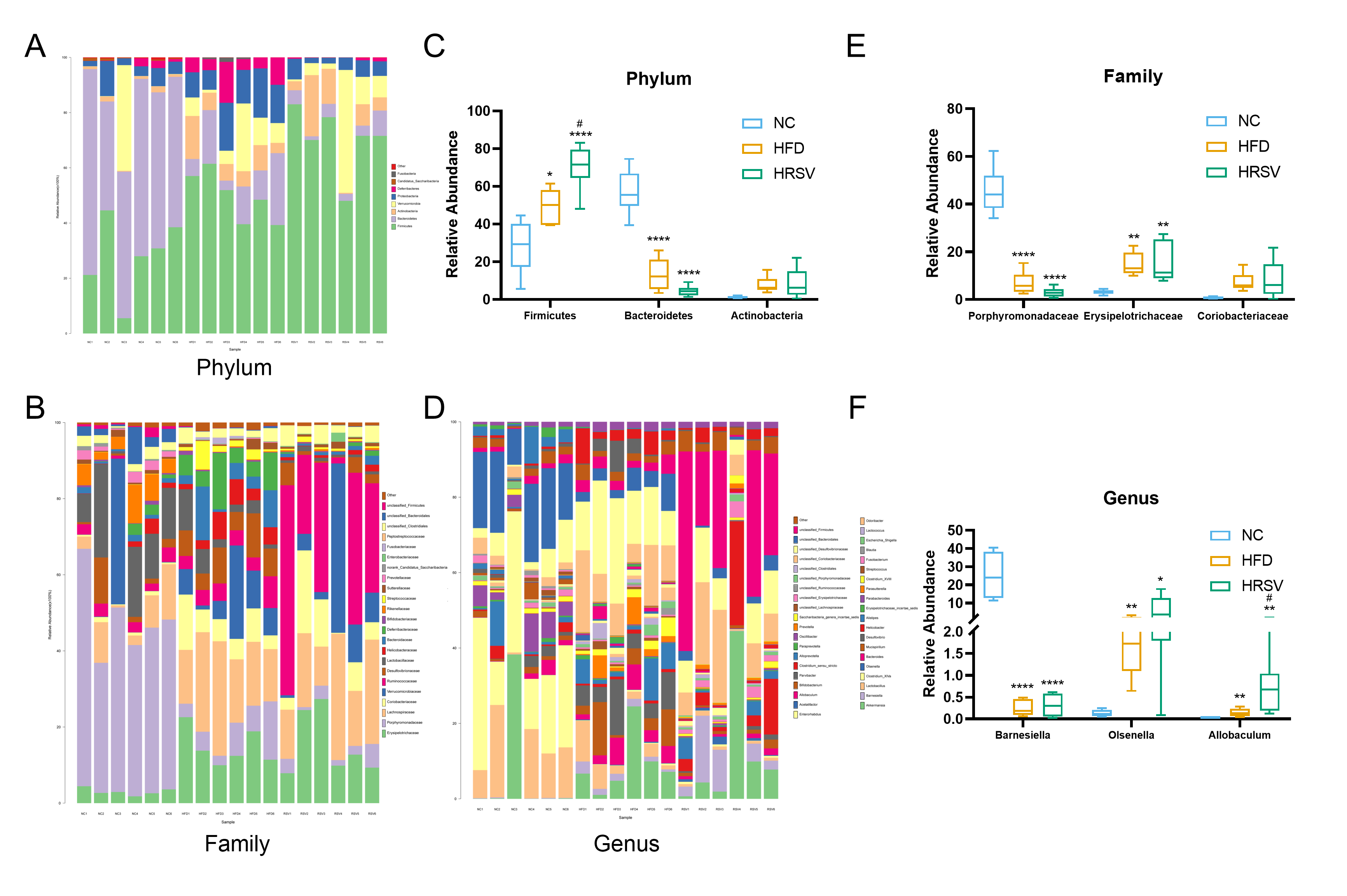


**Figure S3.** High dose RSV caused significant changes in the gut microbiota at the phylum, family, and genus levels. **(A)** Changes in the composition of the gut microbiota in different groups at the phylum level; stacked bar charts represent the relative abundance of major taxa. **(B)** Composition of the gut microbiota at the family level. **(C)** The relative abundance of bacteria differed in the three groups at the phylum level. **(D)** Composition of the gut microbiota at the genus level. **(E)** The relative abundance of bacteria differed in the three groups at the family level. **(F)** The relative abundance of bacteria differed in the three groups at the genus level. Data are shown as the mean±SEM. Variables were analysed using Welch’s test followed by FDR correction. *, p<0.05; **, p<0.01; ****, p<0.0001 vs NC group, #, p<0.05 vs HFD group.


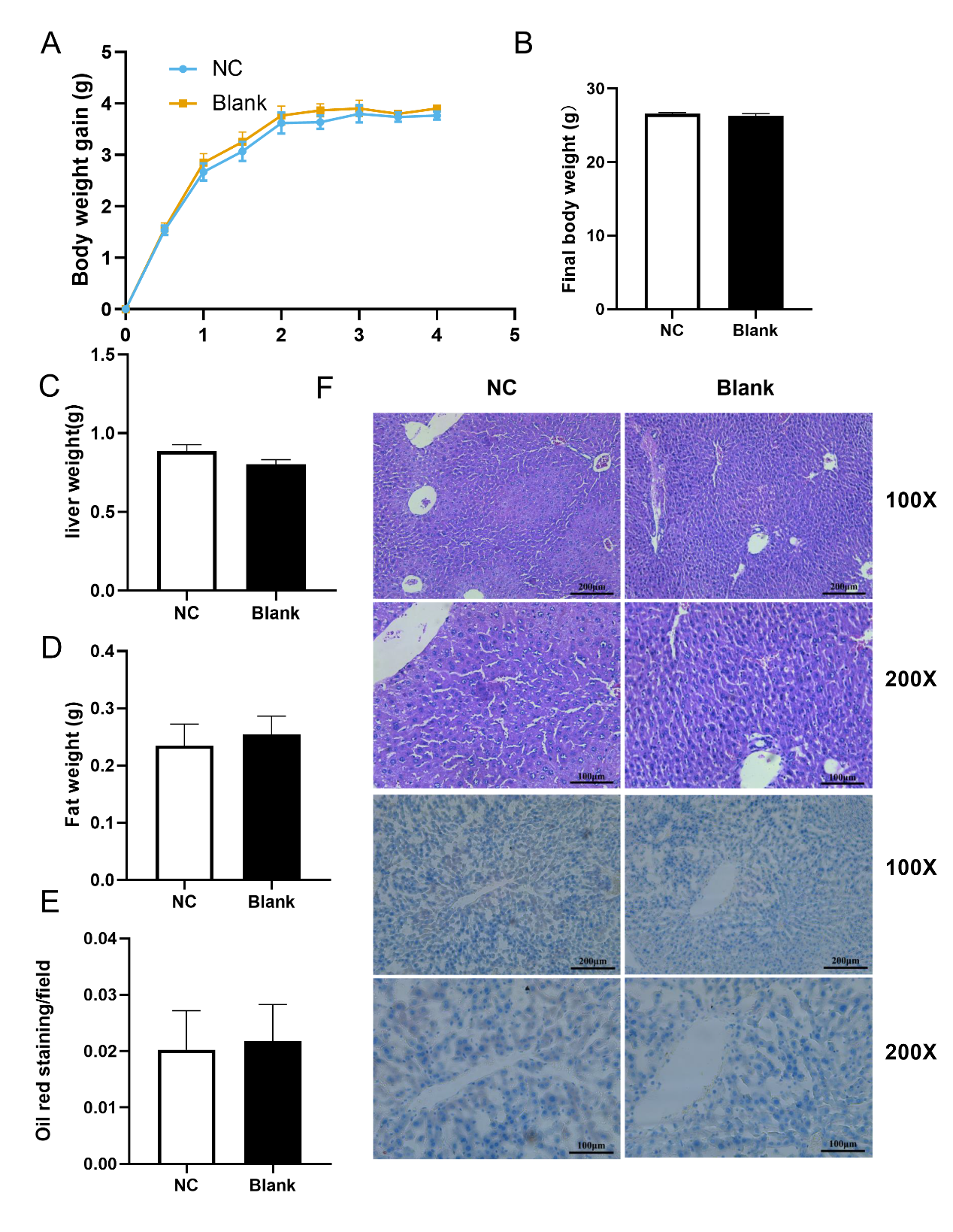


**Figure S4.** CMC had no effects on metabolic parameters in mice. **(A)** Weight gain curve. **(B)** Final body weight. **(C)** Liver triglycerides. **(D)** Fat weight. **(E)** Quantification of the red area/total area by oil red O staining. **(F)** H&E staining and oil red O staining of mouse livers. Data are shown as the mean±SEM, variables were analysed using Tukey’s test or the Mann-Whitney test.
